# Early-Life Respiratory Syncytial Virus (RSV) Infection Triggers Immunological Changes in Gut-Associated Lymphoid Tissues in a Sex-Dependent Manner in Adulthood

**DOI:** 10.3390/cells13201728

**Published:** 2024-10-18

**Authors:** Stella Liong, Felicia Liong, Mitra Mohsenipour, Elisa L. Hill-Yardin, Mark A. Miles, Stavros Selemidis

**Affiliations:** 1 Centre for Respiratory Science and Health, Royal Melbourne Institute of Technology (RMIT) University, Bundoora, VIC 3082, Australia; felicia.liong@rmit.edu.au (F.L.); mark.miles@rmit.edu.au (M.A.M.); 2 School of Health and Biomedical Sciences, Royal Melbourne Institute of Technology (RMIT) University, Bundoora, VIC 3082, Australia; mitra.mohsenipour@rmit.edu.au (M.M.); elisa.hill@rmit.edu.au (E.L.H.-Y.)

**Keywords:** Peyer’s patch, cecal patch, inflammation, respiratory syncytial virus, T cells

## Abstract

Severe respiratory syncytial virus (RSV) infection during early life has been linked to gut dysbiosis, which correlates with increased disease severity and a higher risk of developing asthma later in life. However, the impact of such early-life RSV infections on intestinal immunity in adulthood remains unclear. Herein, we show that RSV infection in 3-week-old mice induced persistent differential natural killer (NK) and T cell profiles within the lungs and gastrointestinal (GI) lymphoid tissues (GALT) in adulthood. Notably, male mice exhibited more pronounced RSV-induced changes in immune cell populations in both the lungs and GALT, while female mice displayed greater resilience. Importantly, early-life RSV infection was associated with the chronic downregulation of CD69-expressing T lymphocytes, particularly T regulatory cells in Peyer’s patches, which could have a significant impact on T cell functionality and immune tolerance. We propose that RSV infection in early life is a trigger for the breakdown in immune tolerance at mucosal surfaces, with potential implications for airways allergic disease, food allergies, and other GI inflammatory diseases.

## 1. Introduction

RSV infection in children is very common, with almost all having encountered it by 2 years of age [1]. While RSV can lead to severe respiratory illness and pneumonia in young children, emerging evidence suggests a connection between severe RSV infections and an increased risk of developing allergic diseases and asthma later in life [2,3]. It is thought that RSV infection during early life, when the lungs and immune system are still maturing, may heighten a child’s susceptibility to developing immunological abnormalities which can lead to chronic illnesses later in life. Epidemiological and preclinical studies have revealed a greater susceptibility in males to severe RSV infection early in life and to the subsequent development of allergic diseases later in life [4,5]. Preclinical studies indicate that while female mice manage RSV infections during the initial phase of infection, male mice experience more severe allergic reactions upon later allergen exposure [5]. Furthermore, RSV-induced wheezing in children has been linked to increased food sensitization within the first year, suggesting a potential relationship between RSV infection and dysregulated gut immunity [6]. However, the impact of early RSV infection on gut immune profiles over time remains unclear.

During viral infections, innate immune cells such as dendritic cells and natural killer (NK) cells play a significant role in shaping the immune response by activating and priming T cells towards distinct T helper (Th)-type responses [7]. In the case of RSV infection, it has been shown that the number of regulatory T cells (Tregs) in the airways and lungs is reduced. Tregs are vital for maintaining immune and allergic tolerance [8]. It has been proposed that the increased severity of RSV disease in males is due to a skewed Th1 anti-viral response leading to an imbalanced Th2/Th17 T cell response, which has been linked with more severe disease outcomes [9,10].

Moreover, NKT cells, which are a subset of T lymphocytes that share characteristics of both the innate and adaptive components of the immune system, are involved in intestinal homeostasis and inflammatory disease progression [11]. However, it is unclear whether RSV infection causes the dysregulation of T cells and NK cells in the gut mucosa.

GALT is a crucial component of gut mucosal immunity, comprising lymphoid follicles in the small intestine (Peyer’s patches) and caecum/appendix (caecal patch) [12]. GALTs are key sites for adaptive immune cell activation and antigen sampling, and thus play an important role in maintaining intestinal tolerance and inflammation. A disruption in GALT function is associated with GI immune pathologies, autoimmunity [12], and even neurological disorders [13,14]. Preclinical studies have shown that early viral exposure, such as gestational influenza A virus infection, can lead to long-term alterations in region-specific GALT immunity, including changes in T cell profiles in offspring [15].

The impact of early-life RSV infection on long-term gut immunity remains unclear. To investigate this, we utilized a preclinical mouse model of early-life RSV infection to examine enduring changes in GALT immunity into adulthood. Our findings reveal that early RSV infection leads to lasting, male-biased immune alterations in both the GALT and the lungs of adult mice, whereas adult female mice showed resilience. These results suggest that the immune changes observed in the gastrointestinal mucosal tissues of males following early RSV infection could be linked to an increased risk of developing allergic diseases later in life.

## 2. Results

### 2.1. Early-Life RSV Infection Drives Persistent Immune Alterations in the Lungs of Adult Male Mice in the Absence of Residual Virus

**Figure 1:**
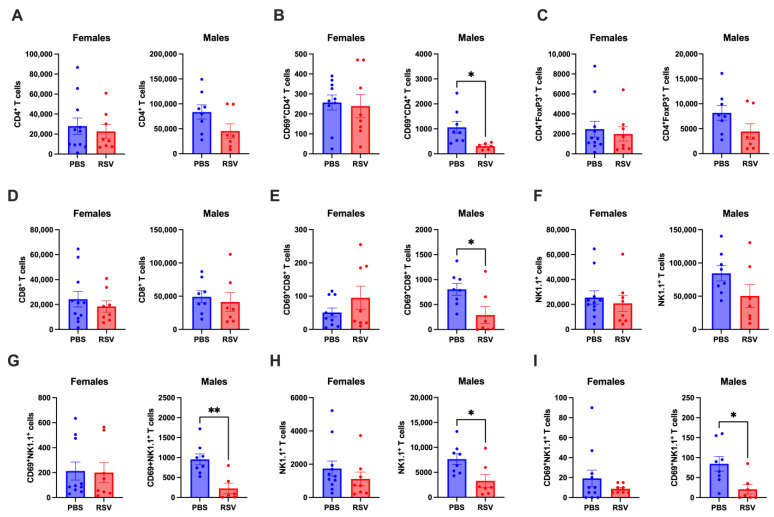
Early-life RSV infection causes chronic immunological changes in the lungs of adult male mice. We employed flow cytometry to measure the absolute numbers of the following cell types in the lungs of 9-week-old mice: (**A**) CD4+ T cells, (**B**) CD69^+^ activated CD4^+^ T cells, (**C**) CD4^+^ regulatory T cells (Tregs), (**D**) CD8^+^ T cells, (**E**) CD69^+^ activated CD8^+^ T cells, (**F**) NK cells, (**G**) CD69^+^ activated NK cells, (**H**) NKT cells, and (**I**) CD69^+^ activated NKT cells. The analysis compared RSV-infected mice (n = 8 females, n = 7 males) with uninfected mice (n = 10 females, n = 8 males) using Student’s *t*-test, with statistical significance set at * *p* < 0.05 and ** *p* < 0.01.

We have previously shown that in adult mice (8–12 weeks old), RSV transcripts can persist in the lungs for up to 42 days following infection with a high RSV dose (2 × 10^7^ PFU) [16]. In this study, we administered a similar high RSV dose to 3-week-old mice to simulate severe RSV infection in young children. In contrast to adult mice, RSV-infected 3-week-old mice did not show persistent RSV transcripts in their lungs at 6 weeks post-infection (Supplementary Figure S1). This discrepancy suggests that age-related immunological differences may affect RSV clearance from the lungs. Despite viral clearance, male mice displayed persistent immune alterations in the lungs following RSV infection, whereas female mice were resilient to these changes (Figure 1). In male mice, the total number of CD4^+^, Tregs (CD4^+^FoxP3^+^) and CD8^+^ T cells remained unchanged (Figure 1A,C,D). However, the numbers of activated (CD69^+^) CD4^+^ and CD8^+^ T cells were significantly reduced (Figure 1B,E). Although the total numbers of NK cells in male mice were unaffected, there was a significant reduction in activated (CD69^+^) NK cells in the lungs (Figure 1F,G). Moreover, both total and activated NKT cell numbers were decreased in the lungs of male mice (Figure 1H,I). These findings indicate that early-life RSV infection causes long-term immune alterations in male mice whilst females were spared of these alterations.

### 2.2. Early Life RSV Infection Does Not Affect GI Tract Anatomy

**Figure 2:**
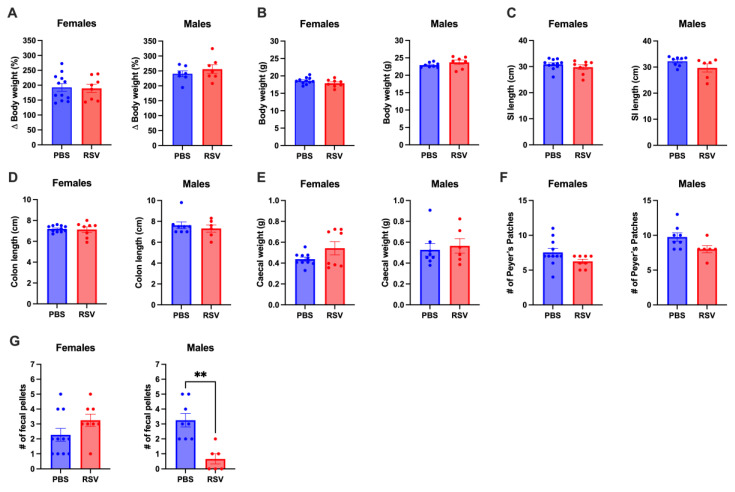
Body weights and gastrointestinal tract analysis. (**A**) Changes in body weight from the day of RSV inoculation (3 weeks old) to the end of the experiment (9 weeks old). (**B**) Body weights measured at 9 weeks of age just before culling. Gastrointestinal (GI) anatomical analyses included: (**C**) lengths of the small intestine (SI), (**D**) lengths of the colon, (**E**) weights of the caecum, (**F**) number of Peyer’s patches, and (**G**) number of formed fecal pellets in the colon at the time of culling. Data were compared between RSV-infected (n = 8 females/group; n = 6 males/group) and uninfected (PBS) mice (n = 11 females/group; n = 8 males/group). Statistical significance was determined using Student’s *t*-test. ** *p* < 0.01.

There were no significant differences in body weight, small intestine lengths, or colon lengths between RSV-infected and uninfected adult mice (Figure 2A–D). RSV infection also did not impact caecal weight or the number of Peyer’s patches (Figure 2E,F). However, male RSV-infected mice had a reduced number of fully formed faecal pellets in the colon, while no such difference was observed in female mice (Figure 2G).

### 2.3. Caecal Patch NK Cells Show Differential Profiles in Male and Female Mice Following Early-Life RSV Infection

**Figure 3:**
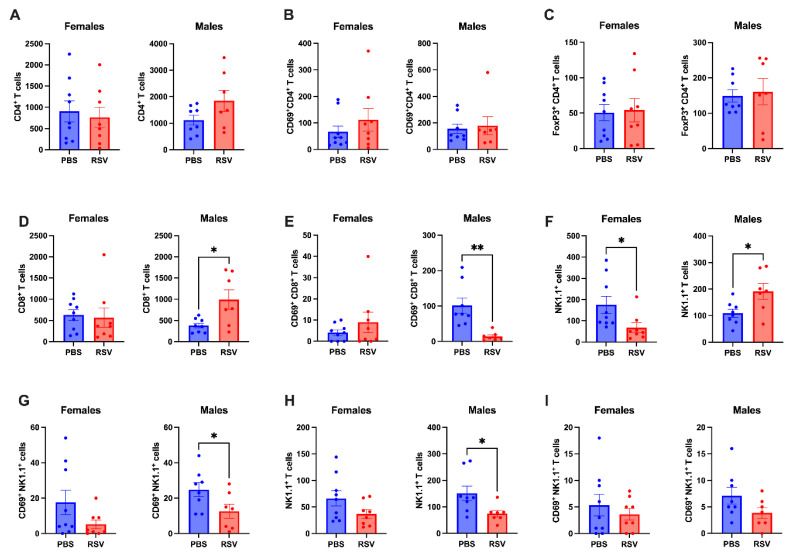
Early-life RSV infection induces persistent differential immune profiles in the caecal patch of adult male mice. We employed flow cytometry to measure the absolute numbers of (**A**) CD4^+^ T cells, (**B**) CD69^+^ activated CD4^+^ T cells, (**C**) CD4^+^ regulatory T cells (Tregs), (**D**) CD8^+^ T cells, (**E**) CD69^+^ activated CD8^+^ T cells, (**F**) NK cells, (**G**) CD69^+^ activated NK cells, (**H**) NKT cells, and (**I**) CD69^+^ activated NKT cells in the cecal patch of 9 week old mice. We compared RSV-infected mice (n = 8 females; n = 7 males) to uninfected mice (n = 10 females; n = 8 males) using Student’s t-test. Statistical significance was indicated as * *p* < 0.05 and ** *p* < 0.01.

We then investigated whether early-life RSV infection affects the immunological profiles in the GALT of adult mice. In the caecal patch, RSV infection did not alter the numbers of CD4+ T cells or regulatory T cells in either male or female mice (Figure 3A–C). While RSV infection did not change the absolute number of CD8+ T cells in the caecal patch of female mice, there was an increase in CD8+ T cell numbers in RSV-infected male mice (Figure 3D). Unexpectedly, activated CD8+ T cells (assessed by CD69 expression) were reduced in the caecal patch of RSV-infected male mice compared to controls (Figure 3E). We also observed sex-dependent differences in NK cell profiles within the caecal patch. RSV infection led to a decrease in NK cells in female mice but an increase in male mice (Figure 3F). Although NK cell activation (CD69+) remained unchanged in female mice after RSV infection, it was significantly reduced in infected male mice (Figure 3G). NKT cell profiles in the caecal patch were unaffected by RSV infection in female mice, but absolute NKT cell numbers were reduced in infected male mice (Figure 3H). The activation of NKT cells did not change in either sex following RSV infection (Figure 3I). Overall, these findings highlight a sex-dependent impact of early-life RSV infection on the caecal patch, with male mice exhibiting dysregulated activation of CD8+ T cells and NK cells.

### 2.4. Chronic Immunological Changes in Peyer’s Patches in Male Mice Following Early Life RSV

**Figure 4:**
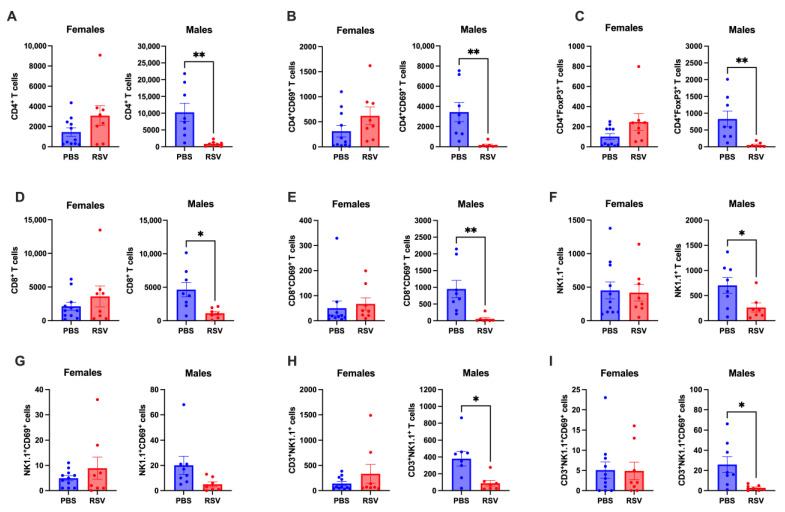
Early-life RSV infection induces persistent differential immune profiles in the Peyer’s patch of adult male mice. Flow cytometry was used to determine the absolute numbers of (**A**) CD4^+^ T cells, (**B**) CD69^+^ activated CD4^+^ T cells, (**C**) CD4^+^ regulatory T cells (Tregs), (**D**) CD8^+^ T cells, (**E**) CD69^+^ activated CD8^+^ T cells, (**F**) NK cells, (**G**) CD69^+^ activated NK cells, (**H**) NKT cells, and (**I**) CD69^+^ activated NKT cells in Peyer’s patches of 9-week-old RSV-infected (n = 8 females; n = 7 males) and uninfected mice (n = 10 females; n = 8 males). Student’s t-test analysis. * *p* < 0.05; ** *p* < 0.01.

Previous studies have shown region-specific immunological changes in GALT following early-life insults in mice [15]. In this study, immune profiling following early-life RSV infection showed a prominent sex bias in Peyer’s patches of adult mice. Female mice were protected from early-life RSV-induced immunological changes in Peyer’s patches, showing no differences between the T cell profiles of infected and uninfected mice (Figure 4). In contrast, RSV-infected male mice showed a reduction in both total and CD69-expressing CD4^+^ and CD8^+^ T cells, as well as Treg cells (Figure 4A–E). Total NK cells were also reduced in infected male mice, although NK cell activation levels remained unchanged (Figure 4F,G). Furthermore, both total and activated NKT cell numbers were decreased in infected male mice (Figure 4H,I). These results suggest that early-life RSV infection leads to greater immune dysregulation in the Peyer’s patches of male mice, while females are largely unaffected.

## 3. Discussion

Despite the evidence that severe RSV infection can cause dysregulation of the GI microbiome, its long-term effects on GI immunity remain poorly understood. Some studies have suggested that children with RSV-induced wheeze may have an increased risk of food sensitisation within the first year of life [6], hinting at a potential link between early-life viral infections and later allergic conditions. Herein, we show that early-life RSV infection leads to a persistent immune system dysregulation in both the lungs and GALT of male mice, while female mice appear to be largely protected. Thus, these findings demonstrate that male mice are more susceptible to persistent T and NK cell dysregulation in mucosal tissues following early-life RSV infection, which may contribute to exacerbated allergic responses later in life.

Some studies have explored the lung–gut axis during viral infections and the impact of gut dysbiosis on the development of airway allergic diseases [17]. However, the long-term effects of early-life RSV infection on gut immunity remain unclear. The pathological activation of T cells can result in the breakdown of immune tolerance and intestinal inflammation. In male mice, CD69^+^CD8^+^ T cells in caecal and Peyer’s patches, and CD69^+^CD4^+^ T cells in Peyer’s patches were reduced with RSV infection compared to uninfected controls. CD69 deficiency has been linked to enhance immune allergic responses that exacerbate asthma and contact dermatitis in experimental models [18]. For example, the adoptive transfer of CD69-deficient CD4^+^ T cells into RAG^-/-^ mice (deficient in lymphocytes) induced severe colitis due to the increased secretion of proinflammatory cytokines (IFN-γ, TNF-α, IL-21) and decreased levels of anti-inflammatory cytokines (TGF-β1) [19]. RSV infection is known to reduce regulatory T cells (Tregs) in the airways and lungs, which play an important role in maintaining immune and allergic tolerance [8]. In the GI mucosa, Tregs specific to food allergens promote intestinal tolerance by suppressing food sensitisation [20]. Treg-deficient mice display poor control of food antigen uptake, lymphoproliferation, increased IgE levels, and eosinophilia [21]. Although beyond the scope of this study, future studies should investigate whether early-life RSV infection reprograms GALT T cell effector functions, potentially influencing the development of colitis or allergic disorders later in life.

Following the resolution of infection, effector T cell populations retract, and a small proportion differentiate into memory T cells. A subset of memory T cells known as tissue-resident memory T (T_RM_) cells permanently reside in the peripheral organs. CD69 is also a canonical marker of T_RM_ cells, and so GALT T cells that express CD69 in this study could be representative of T_RM_ cells. The local microenvironment within peripheral organs, including the GI tract, can regulate CD69 expression to promote the survival and retention of T_RM_ cells [22]. Given that RSV is cleared from the lungs within 10 days of infection [23], and the mice were allowed to recover for 6 weeks, the observed downregulation of CD69-expressing CD8^+^ T cells in the Peyer’s patches might reflect a retracted CD8^+^ T_RM_ pool. Further studies are required to investigate whether early-life RSV infection is associated with the increased cell death of pre-existing GALT T_RM_ cells. Furthermore, a retracted CD8^+^ T_RM_ population could potentially skew the gut immune response from a Th1 to a Th2 profile. This shift might reduce protection against future viral infections, which require Th1 responses, while increasing the risk for allergic diseases mediated by Th2 responses. Chronic Th2 immune responses to RSV infection have been associated with driving the development of allergic complications later in life [3], and since food allergies are typically Th2-driven, early-life RSV infection could contribute to an increased risk of detrimental food sensitisation.

Gut microbiota diversity varies by sex, with females generally exhibiting greater α-diversity (bacterial species richness and evenness) compared to males [24]. Infants with severe RSV infections tend to have slightly lower α-diversity in their gut microbiomes than those with moderate RSV infections or healthy controls [25]. Additionally, patients with food allergies often show reduced α-diversity compared to non-allergic individuals [26]. Further studies are needed to determine whether our early-life RSV infection model recapitulates this reduced α-diversity. Mechanistically, studies have linked Treg cell deficiency to decreased gut microbiota diversity [27]. Mice deficient in T cells exhibited reduced gut microbial diversity compared with wild-type animals and this was rescued by the adoptive transfer of Foxp3^+^ Treg cells [28]. These findings suggest that early-life severe RSV infection may alter gut microbiome composition and that the increased susceptibility of males to severe RSV infection and subsequent allergic diseases could be related to reduced gut microbial diversity and its impact on T cell immune responses.

NKT cells are a distinct subset of T cells that have been shown to contribute to gut immune responses in health and disease [11]. They can be divided into two main types: Type I NKT cells, which are activated by glycolipid antigens and can produce a range of cytokines, including Th1 (IFN-γ, TNF-α) and Th2 (IL-4, IL-10), depending on environmental cues; and Type II NKT cells, which recognize self-lipids and can both promote inflammation and inhibit Type I NKT cell activation [29,30]. CD69-expressing NKT cells, which resemble tissue-resident memory (TRM) cells, are implicated in allergic conditions like atopic dermatitis [31]. Further work is needed to elucidate the role of NKT_RM_ cells in gut health and the implications of their reduced numbers in the GALT of male mice following early-life RSV infection. NK cells, as innate immune cells, are crucial for antiviral defense and T cell regulation. They also serve as a significant source of type II IFNs, which promote Th1 immune responses [7]. In this study, male mice infected with RSV exhibited a reduced number of CD69+ NK cells compared to uninfected controls, suggesting impaired NK cell activation. This reduction in NK cell activity could hinder the effectiveness of the Th1 immune responses and compromise CD8+ T cell function in these RSV-infected males.

Sexual dimorphism has been shown to dictate the pathogenesis of respiratory viral infections [5,32,33]. It is thought that male sex hormones and the regulation of androgen receptors (AR) on Th2 immunity influences susceptibility to allergic diseases following viral infections. It has been proposed that this susceptibility to severe RSV disease in males is due to a skewed Th1 anti-viral response and a dysregulated Th2/Th17 T cell response [9,10]. In a preclinical model of neonatal RSV infection, female pups have better viral control and elevated IFN-β levels compared to males [5]. This difference in the immune response in male pups promoted increased Th2 and Th17 cells and IL-33 production (Th2 cytokine), and was correlated with the exacerbation of allergic responses. In sexually mature male mice, elevated testosterone levels and AR activity are associated with a downregulation of Th2- and Th17-mediated airway inflammation [32]. This may explain why asthma prevalence is lower in males compared to females during adulthood. A more in-depth analysis is warranted to investigate how testosterone and AR activity regulate gut immunity during viral infections.

Our manuscript has several limitations, including a focussed analysis of RSV virus detection in the GALT using PCR, along with the assessment of Th1 and Th2 mediators—specifically, IFN-γ, TNF-α, and Type I IFN for Th1, and IL-10 for Th2. Additionally, this analysis should include the nasal compartments, particularly regarding IL-10, as its levels in this tissue have been shown to negatively correlate with asthma onset [34]. In children, low IL-10 production in the nasal passages during viral infections is associated with asthma development by age 6. It would be valuable to investigate whether IL-10 levels are also reduced in the GALT following RSV infection. Another limitation is that we did not analyse gut tissue using H&E staining or immunofluorescence. This aspect warrants a separate study to address the pathological implications of early RSV infection, particularly in the context of colitis inflammatory disease models, such as the dextran sulphate sodium (DSS) model or the IL-10 knockout mouse model. The connection between IL-10, asthma onset, and colitis susceptibility in this context merits further investigation. Finally, it is important to explore pre-infection factors that influence the immune response before an infection occurs. In the context of RSV infection, several pre-infection factors play a role, especially regarding how different sexes respond to the virus. Future studies should particularly consider Thymic Stromal Lymphopoietin (TSLP), an epithelial-cell-derived cytokine that helps shape immune responses by promoting Th2 cell differentiation. Differences in TSLP levels between sexes could significantly contribute to variations in immune responses to RSV.

In conclusion, early life RSV infection leads to lasting immunological changes in the GALT that persist into adulthood. Male mice exhibited a greater susceptibility to chronic immune dysregulation in the gut following RSV infection compared to females. Further research is needed to explore the potential sex-dependent differences in gut immune responses during active RSV infection. Gaining a deeper understanding of how early-life RSV and similar viral infections impact mucosal immunity in the gut could inform the development of targeted therapies to prevent chronic allergic and inflammatory diseases.

## 4. Materials and Methods

### 4.1. Animal Ethics

This study received approved from the Royal Melbourne Institute of Technology University (RMIT) Animal Ethics Committee (AEC# 24336), adhering to the guidelines outlined in the Australian Code of Practice for the Care of Experimental Animals and National Health and Medical Research Council of Australia (NHMRC).

### 4.2. Respiratory Syncytial Virus (RSV)

Human respiratory syncytial virus (RSV A long strain) was propagated as previously described using HEp-2 cells by for 3–4 days until at least an 80% cytopathic effect was observed [16]. Viral titres were determined by plaque assay using HEp-2 cells.

### 4.3. RSV Inoculation of Mice

Time-mated C57BL6 female mice were purchased from the Animal Resource Centre (Perth, Australia). At postnatal day 21 (3 weeks old), mice from n = 7 dams were weaned and randomly assigned within each litter (to control for litter effects) to receive either an intranasal inoculation of RSV A long strain at 2 × 10^7^ plaque-forming units (PFU) or a mock infection with PBS. Mice were provided with standard mouse chow diet and water ad libitum. At 6 weeks post-infection (when the mice were 9 weeks old, in adulthood), they were euthanized for tissue collection. The gut was carefully dissected, and measurements were taken for the length of both the small intestine and colon. Additionally, cecal weights, the number of Peyer’s patches, and the quantity of formed fecal pellets in the colon were recorded.

### 4.4. RNA Extraction and qPCR

RNA was extracted from lung tissue as previously described [35]. Total RNA was extracted from the homogenate using the RNeasy Mini kit (Qiagen, Clayton, Australia). The High-Capacity cDNA Reverse Transcription Kit (Applied Biosystems, CA, USA) was used as per manufacturer’s instruction to convert 1–2 μg of RNA into cDNA. The expression of relevant genes was evaluated using pre-designed TaqMan primers (Life Technologies, Waltham, MA, USA) and the TaqMan Universal PCR Master Mix (Applied Biosystems, Waltham, MA, USA). RSV titres were measured using custom-designed primers for the F gene: 5′-TTGGATCTGCAATCGCCA-3′, 5′-CTTTTGATCTTGTTCACTTCTCCTTCT-3′ using Fast SYBR Green PCR Master Mix (Applied Biosystems). Gene quantitation was performed in triplicate as previously described and analysed using the comparative Ct method, with target gene expression normalised against the housekeeping gene RPS18 and the uninfected PBS group set to 1 [35].

### 4.5. Flow Cytometry

Tissue samples from the lung (largest right lobe), cecal patch, and Peyer’s patch were processed into single-cell suspensions as previously described [15,35]. In brief, the tissues were mechanically dissociated and then incubated with 0.1% liberase at 37 °C for 30 min (for GALT) or 45 min (for lungs). For the lungs, following enzymatic digestion, cells were pushed through a 18 G needle five times and then through a 21 G needle five times to ensure complete dissociation. Both GALT and lung samples were then filtered through a 40 µm strainer, centrifuged at 400× *g* and washed with FACS buffer (2.5% FBS in PBS). Cells were then incubated for 2–3 min at room temperature in a red blood lysis buffer (2–3 min at room temperature) to remove any contaminating erythrocytes in the samples and then washed with an FACS buffer. Cells were then resuspended in an ice-cold FACS buffer ready for staining for flow cytometry following established protocols (14). Cells were labeled using the Live/Dead Fixable Aqua Dead Cell Stain Kit, an Fc block antibody, and the following surface antibodies (BioLegend, San Diego, CA, USA) for 25 min at 4 °C: CD45-Alexa Fluor 700 (clone 30-F11), CD3-APC (clone 145-2C11), CD8-PerCP-Cy5.5 (clone 53.6.7), CD4-BV605 (clone RM4-5), Ly6C-FITC (clone HK1.4), Ly6G-APC-Cy7 (clone 1A8), CD11b-BV421 (clone M1/70), NK1.1-BV605 (clone PK136), and CD69-BV650 (clone BM8). For intracellular staining, cells were fixed and permeabilized using the Foxp3/Transcription Factor Staining Buffer Set (eBioscience, Waltham, MA, USA) and then stained with FoxP3-PE antibody (clone FJK-16s; eBioscience) for 15 min at room temperature. Cell analysis was performed using the LSRFortessa™ X-20 (BD Biosciences), and data were analyzed with FlowJo v10 software. Cell counts were expressed as total cells per lung, cecal patch or Peyer’s patch. The gating strategy for immune cell populations is illustrated in Supplementary Figure S2.

### 4.6. Statistical Analysis

Graphs were generated using GraphPad Prism software v10 (San Diego, CA, USA). All data are expressed as mean +/− SEM. Comparisons between the two groups were performed using Student’s *t*-test. *p* < 0.05 was considered statistically significant.

## Data Availability

Data will be made available upon reasonable request.

## Supplementary Materials

The following supporting information can be downloaded at: https://www.mdpi.com/article/10.3390/cells13201728/s1, Figure S1: Gating strategy for flow cytometry analysis.; Figure S2: Residual RSV is not detectable in the lungs of adult mice following early life RSV infection.
