# Supplementary figures and images for: Early-Life Respiratory Syncytial Virus (RSV) Infection Triggers Immunological Changes in Gut-Associated Lymphoid Tissues in a Sex-Dependent Manner in Adulthood
Source: Cells. 2024 Oct 18;13(20):1728. doi: 10.3390/cells13201728 (PMC11506009; doi:10.3390/cells13201728)

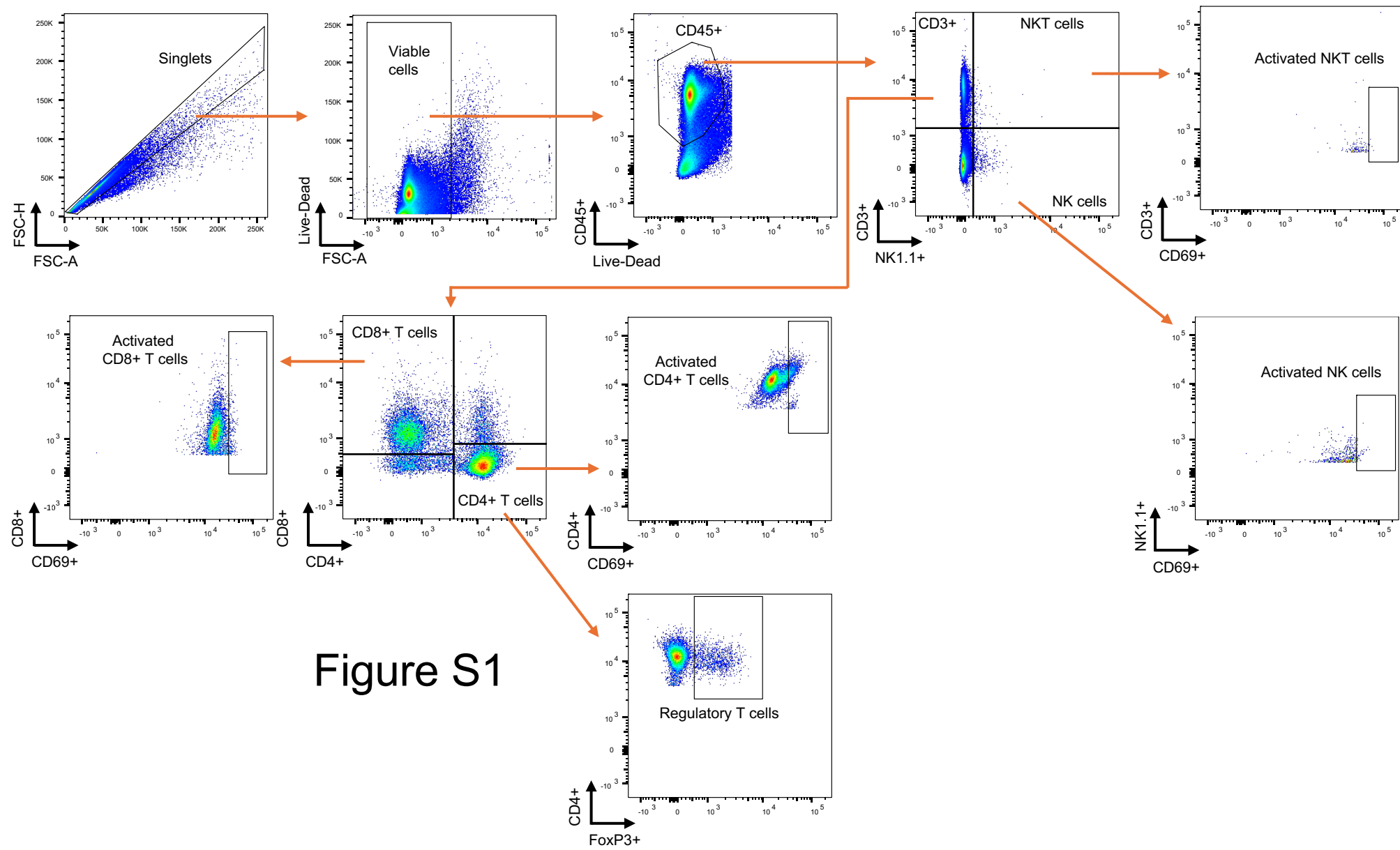

Figure S2

## Females

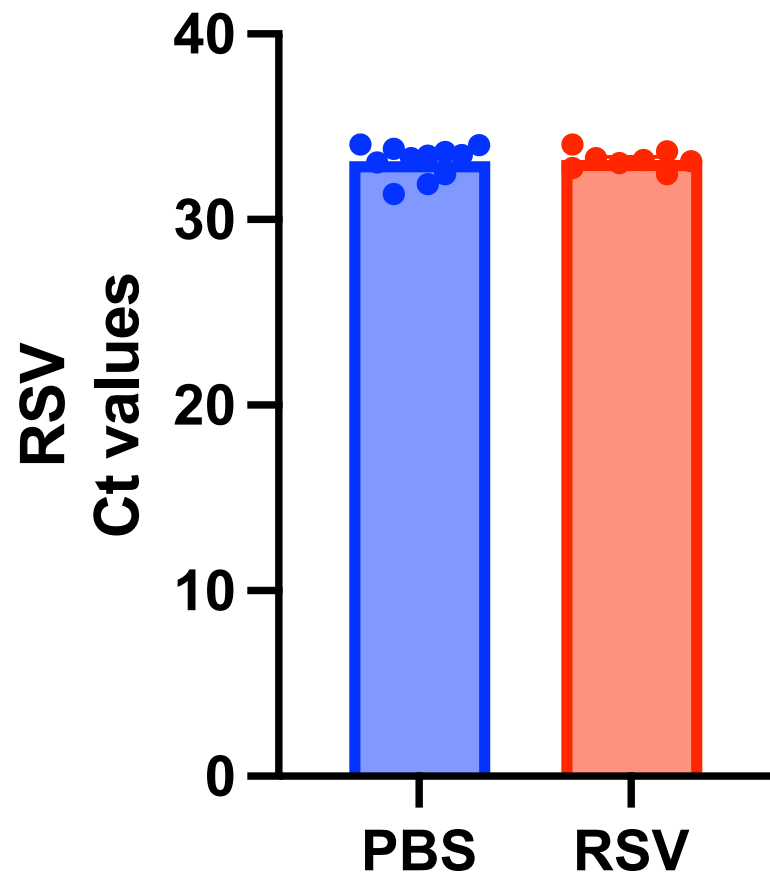

## Males

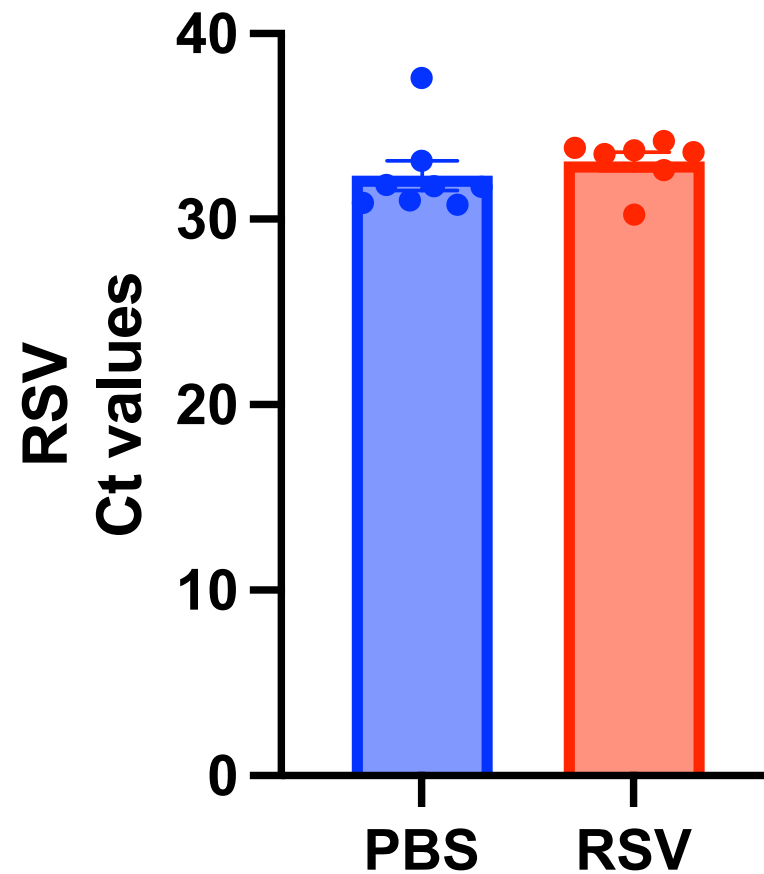

Supplement: Supplementary file 1 [file cells-13-01728-s001.zip › cells-3226219-supplementary.pdf]
